# Poor outcome following percutaneous balloon mitral valvotomy in patients with atrial fibrillation

**DOI:** 10.15171/jcvtr.2016.26

**Published:** 2016-09-30

**Authors:** Naser Aslanabadi, Samad Ghaffari, Naser Khezerlouy Aghdam, Masoumeh Ahmadzade, Babak Kazemi, Babak Nasiri, Ahmad Separham, Bahram Sohrabi, Mohamadreza Taban, Arash Aslanabadi

**Affiliations:** Cardiovascular Research Center, Tabriz University of Medical Sciences, Tabriz, Iran

**Keywords:** Mitral Stenosis, Balloon Mitral Valvotomy, Atrial Fibrillation

## Abstract

***Introduction: ***Atrial fibrillation (AF) is the most common arrhythmia in patients with mitral stenosis (MS) and it may increase complications and decreases success rates of percutaneous balloon mitral valvotomy (PBMV). This study aimed to investigate the short and long term results of PBMV in patients with AF compared to sinus rhythm (SR).

***Methods:*** In this cross sectional study, 1000 patients with MS who had undergone PBMV between 1999 and 2013 were enrolled including 585 and 415 patients with AF and SR respectively. Patients were followed for a mean of 7.27 ± 3.16 years. Clinical, echocardiographic and hemodynamic data were collected. Procedure success, in-hospital and long-term outcome were evaluated.

***Results: ***Patients with AF were older and had greater symptoms, mitral regurgitation, mitral echocardiographic score, and mitral pressure gradient before PBMV. PBMV success rate were significantly lower in AF group (*P* < 0.001). In-hospital complications, including severe mitral regurgitation, emergency mitral valve surgery, peripheral embolism and long-term complications, including mortality, re-valvotomy, mitral replacement surgery and peripheral embolism/stroke were significantly higher in patients with AF.

***Conclusion: ***AF leads to worse in-hospital and long-term outcome and lower PBMV success rate. Repeated assessment and early decision to PBMV in patients with MS to reduce AF and AF related complication seems necessary.

## Introduction

Mitral stenosis (MS) is still a public health problem in developing countries.^1,2^ Percutaneous balloon mitral valvotomy (PBMV) is performed as preferred alternative to surgery for most patients with hemodynamically significant MS.^3-9^ Atrial fibrillation (AF) is the most common arrhythmia that occurs in 40%-75% of symptomatic patients with MS.^10,11^ Several studies have shown that AF affects short and long term outcomes and the mortality risk of PBMV and in most cases it has been associated with a decreased success rate of PBMV and a worse clinical outcome.^12-18^ So considering that the previous studies have been conducted on a smaller number of patients, this study aimed at investigating the immediate success rates of PBMV, short and long term results, and mortality risk in patients with MS and AF compared to sinus rhythm (SR) in a large data base.

## Materials and Methods

Between 1999 and 2013, about 1800 patients underwent PBMV at our tertiary heart center. Out Of these, 1000 consecutive patients who had complete information requirements on their files were enrolled in this cross sectional study. All patients were divided into two groups based on initial cardiac rhythm (SR vs. AF). In each group, demographic variables such as age, gender and clinical variables, including the severity of dyspnea according to New York Heart Association functional class (NYHA-FC), echocardiographic variables including MS echocardiographic score, and the mitral valve area (MVA) before and after PBMV and hemodynamic variables such as mean left atrial pressure (LAP), mean transmitral pressure gradient (mTMPG) and pulmonary artery systolic pressure (sPAP) levels were checked and the results were compared between the two groups. In-hospital complications including emergent mitral valve replacement (MVR), developing severe mitral regurgitation (MR≥3⁺), tamponade, death, and peripheral embolism, as well as adverse events at long-term follow-up such as death, peripheral embolism/stroke, and need for re-PBMV or MVR were checked and compared in the two groups. Demographic clinical and procedural variables were collected retrospectively using a review of medical records and telephone contact. Our technique in performing PBMV (Inoue technique) has been reported previously.^19,20^ PBMV success was defined as a final MVA>1.5 cm^2^ without resulting >2+ angiographic MR.^21^ In all patients transthoracic echocardiography was done at least one week prior to the procedure and transesophageal echocardiography was performed on the morning of the procedure and echocardiographic score of the mitral apparatus, baseline MVA, left ventricular ejection fraction (LVEF) and presence of MR were determined. Mitral valvular and sub-valvular morphology was graded according to the Wilkins’s scoring system which assigns higher scores to values with more severe disease.^22^ MVA was calculated by planimetry or, in the absence of significant mitral regurgitation, from pressure half-time.^23^ Semi-quantitative estimation of mitral regurgitation was made with color flow mapping in parasternal long axis and apical four-chamber views. mTMPG and sPAP were estimated using continuous-wave Doppler echocardiography. LAP and mTMPG were measured during catheterization. All PBMV procedures were done without heparin.^24^

### Statistical analysis

Categorical variables, expressed as percentages, were compared by Fisher or χ^2^ exact tests. Continues data, shown as mean ± SD, were compared by Mann-Whitney U test and independent sample t test. Multivariate regression analysis was performed to determine the independent variables for predicting adverse events and subgroup analysis. *P* values <0.05 were considered to indicate statistical significance. Data recording and analysis were performed using SPSS version 17.0 software (SPSS Inc., Chicago, IL).

## Results

### Baseline characteristics

Out of 1000 patients who were enrolled in the study, 239 were male (23.9%) and 761 were female (76.1%), aged 14-78 years (mean age 49.15 ± 12.98 years). Five hundred eighty-five patients had SR and the rest 415 were in AF. Table 1 shows the base findings in both groups. The AF group was older, was more in dyspnea NYHA-FC (III-IV), had greater MR before PBMV, higher mitral valve echo scores, and higher mTMPG compared to the SR group. sPAP, MVA, and LAP were similar in both groups. Table 2 shows the findings of two groups after PBMV. There were more cases of severe MR (MR≥ 3^+^) and dyspnea NYHA-FC (III-IV), higher LAP, higher mTMPG and smaller MVA in the AF group compared to the SR group. sPAP was similar in both groups. Three patients with AF converted to SR after PBMV.

**Table 1:** Baseline characteristic in both groups

	** SR (n=585) **	**AF (n=415)**	** P value**
Age, years	45.42 ± 12.08	54.40±12.39	<0.001^a^
Female	454 (77.6%)	307 (74%)	0.18
Valve score	8.37±1.18	8.77±0.96	<0.001^a^
NYHA-FC III-IV	198 (33.8%)	180 (43.4%)	0.002^a^
No MR	156 (26.7%)	63 (15.2%)	<0.001^a^
MR +1	258 (44.1%)	137 (33%)	<0.001^a^
MR +2	171 (29.2)	215 (51.8%)	<0.001^a^
sPAP, mm Hg	45.43±12.13	54.66±12.88	0.89
MVA, cm^2^	0.96±0.19	0.95±0.20	0.35
LAP, mm Hg	28.41±8.60	28.73±6.37	0.52
mTMVG, mm Hg	11.80±2.48	12.27±2.96	0.006^a^

Abbreviations: AF, atrial fibrillation; LAP, left atrial pressure; MR, mitral regurgitation; mTMVG, mean transmitral valve gradient; MVA, mitral valve area; NYHA-FC, New York Heart Association functional class; sPAP, systolic pulmonary artery pressure; SR, sinus rhythm; Values were shown as mean ± SD. ^a^ *P* value is significant.

**Table 2:** Post-PBMV results and in-hospital outcome

	** SR (n=585) **	** AF (n=415) **	**P value**
sPAP, mmHg	36.25±5.58	35.83±5.56	0.24
MVA, cm^2^	1.80±0.24	1.62±0.42	0.003^a^
LAP, mmHg	17.19±4.37	18.26±5.23	<0.001^a^
mTMVG, mm Hg	4.62±2.31	5.19±2.69	<0.001^a^
All complications	20 (3.4%)	45 (10.8%)	<0.001^a^
MVR	10 (1.7%)	23 (5.5%)	0.001^a^
Severe MR (≥3⁺)	0	4 (1%)	0.02^a^
Tamponade	0	1 (0.2%)	0.41
Peripheral emboli	10 (1.7%)	17 (4.1%)	0.02 ^a^

Abbreviations: AF, atrial fibrillation; LAP, left atrial pressure; MR, mitral regurgitation; mTMVG, mean transmitral valve gradient; MVA, mitral valve area; MVR, mitral valve replacement; sPAP ,systolic pulmonary artery pressure; SR, sinus rhythm; Values were shown as mean±SD. ^a^ *P* value is significant.

### Procedure success

PBMV was successful in 554 (94.7%) of SR patients and 281 (67.7%) of AF patients (*P* < 0.001). After PBMV, mTMPG were significantly lower and MVA were significantly higher in SR group. In subgroups analysis we evaluated the relationship between demographic and baseline characteristic with incidence of complications in both groups. In the AF group (Table 3), older age, severe dyspnea NYHA-FC (III-IV) before PBMV, MR before and after PBMV and higher LAP before PBMV had a significant relationship with the incidence of complications during follow-up. Multivariate regression analysis showed that NYHA-FC before PBMV could independently predict complications during follow-up (odds ratio [OR]: 1.66, 95% CI: 1.002-2.969; *P *= 0.04). Also in the SR group (Table 4), MR before and after PBMV, the success rate of the procedure, the mitral valve echocardiographic score and MVA before and after PBMV were significantly associated with adverse events rate. Multivariate regression analysis showed that the low MVA after PBMV, could independently predict occurrence of late complications in this group (OR: 4.42, 95% CI: 1.022-1.108; *P *= 0.04).

**Table 3:** Comparison of baseline and echocardiographic findings between complicated and uncomplicated patients in the SR group

	** Complicated (n=84) **	**Uncomplicated (n=501)**	** P value**
Age, years	47.79±14.13	45.02±11.68	0.052
Female	687 (81%)	386 (77%)	0.42
NYHA-FC I-II before PBMV	57 (67.9%)	330 (65.9%)	0.7
NYHA-FC III-IV before PBMV	27 (32.1%)	171 (34.1%)
NYHA-FC I-II after PBMV	72 (85.7%)	453 (90.4%)	0.18
NYHA-FC III-IV after PBMV	12 (14.3%)	48 (9.6%)
MR before PBMV	82 (97.6%)	347 (69.3%)	<0.001a
Absent MR before PBMV	2 (2.4%)	154 (30.7%)
MR after PBMV	82 (97.6%)	268 (53.5%)	<0.001a
Absent MR after PBMV	2 (2.4%)	233 (46.5%)
PBMV success	69 (82.1%)	485 (96.8%)	<0.001a
Valve score	8.11±1.12	8.41±1.18	0.03a
sPAP before PBMV, mmHg	45.79±9.53	45.95±9.68	0.89
sPAP after PBMV, mmHg	35.8±5.78	38.23±5.55	0.43
MVA before PBMV, cm^2^	0.9±0.19	0.97±0.18	0.001a
MVA after PBMV, cm^2^	1.66±0.27	1.82±0.22	<0.001a
LAP before PBMV, mmHg	29.11±7.92	28.30±8.71	0.42
LAP after PBMV, mmHg	17.26±4.66	17.18±4.32	0.87
mTMPG before PBMV, mm Hg	12.02±2.35	11.76±2.50	0.37
mTMPG after PBMV, mm Hg	4.51±2.40	4.64±2.2	0.61

Abbreviations: LAP, left atrial pressure; MR, mitral regurgitation; mTMVG, mean transmitral valve gradient; MVA, mitral valve area; NYHA-FC, New York Heart Association functional class; PBMV, percutaneous balloon mitral valvotomy; sPAP, systolic pulmonary artery pressure; SR, sinus rhythm; Values were shown as mean±SD. ^a^*P* value is significant.

**Table 4:** Comparison of baseline and echocardiographic findings between complicated and uncomplicated patients in the AF group

** Complicated (n=139) **	**Uncomplicated (n=280)**	** P value**
Age, years	57.38±13.00 5	2.90±11.80	<0.001^a^
Female	101 (72.7%)	206 (74.6%)	0.66
NYHA-FC I-II before PBMV	91 (65.5%)	144 (52.2%)	0.01^a^
NYHA-FC III-IV before PBMV	48(34.5%)	132 (47.8%)
NYHA-FC I-II after PBMV	115(82.7%)	237 (85.9%)	0.44
NYHA-FC III-IV after PBMV	24 (17.3%)	39 (14.1%)
MR before PBMV	134 (96.4%)	218 (79%)	<0.001^a^
Absent MR before PBMV	5 (3.6%)	58 (21%)
MR after PBMV	129 (92.8%)	189 (68.5%)	<0.001^a^
Absent MR after PBMV	10 (7.2%)	87 (31.5%)
PBMV success	93 (66.9%)	188 (68.1%)	0.8
Valve score	8.77±1.03	8.76±0.93	0.93
sPAP before PBMV, mmHg	47.6±9.32	45.48±9.95	0.12
sPAP after PBMV, mmHg	34.79±5.41	36.35±5.58	0.007^a^
MVA before PBMV, cm^2^	0.93±0.18	0.96±0.21	0.09
MVA after PBMV, cm^2^	1.59±0.39	1.63±0.43	0.36
LAP before PBMV, mmHg	29.69±6.64	28.25±6.18	0.02^a^
LAP after PBMV, mmHg	18.86±4.5	18.06±5.56	0.26
mTMPG before PBMV, mmHg	12.31±2.90	12.263.0±	0.85
mTMPG after PBMV, mmHg	5.23±0.64	5.17±2.72	0.84

Abbreviations: AF, atrial fibrillation; LAP, left atrial pressure; MR, mitral regurgitation; mTMVG, mean transmitral valve gradient; MVA, mitral valve area; NYHA-FC, New York Heart Association functional class; PBMV, percutaneous balloon mitral valvotomy; sPAP, systolic pulmonary artery pressure; Values were shown as mean±SD. ^a^ *P* value is significant

### In-hospital (short-term) outcome

Table 2 shows the in-hospital results in two groups. In total, 45 patients of AF group (10.8%) and 20 patients of SR group (3.4%) had in-hospital complications (*P* < 0.001). MVR surgery was required in 23 (5.5%) of AF patients and in 10 (1.7%) of the SR group (*P *< 0.001). Severe MR occurred in 4 (1%) patients in the AF group and in none of the SR group (*P *= 0.02). Tamponade occurred in 1 (0.2%) patient in the AF group and in none of the SR group (*P *= 0.41). Peripheral embolic events occurred in 17 (4.1%) patients in the AF group and in 10 (1.7%) of SR patients (*P *= 0.02).

### Long-term outcome

Table 5 show long-term follow-up results of patients in both groups. Patients were followed for a mean of 7.27±3.16 years (a minimum of 2 years and a maximum of 14 years). Adverse events (including MVR, re-PBMV, stroke, peripheral embolism and death) were seen in 148 (35.7%) patients in the AF group and in 93 (15.9%) patients in the SR group (*P* < 0.001). MVR was required in 31 (7.5%) patients in the AF group and 19 (3.2%) patients in the SR group (*P* < 0.003). re-PBMV was performed in 77 (18.6%) patients in the AF group and in 46 (7.9%) patients in the SR group (*P* < 0.001). Stroke occurred in 17 (4.1%) patients with AF and in 11 patients (1.9%) with SR (*P *= 0.03). Peripheral embolic events occurred in 12 patients (2.9%) in the AF and in 8 (1.4%) patients in the SR group (*P *= 0.03). Twenty-two (5.3%) patients in the AF and 7 (1.2%) in the SR group died during long term follow up (*P* < 0.001).

**Table 5:** Long term outcome in both groups

	** SR (n=585) **	** AF (n=415) **	** P value**
All complications	93 (15.9%)	148 (35.7%)	<0.001^a^
MVR	19 (3.2%)	31 (7.5%)	0.003^a^
re-PBMV	46 (7.9%)	77 (18.6%)	<0.001^a^
CVA	11 (1.9%)	17 (4.1%)	0.03^a^
Peripheral emboli	8(1.4%)	12 (2.9%)	0.09
Mortality	7 (1.2%)	22 (5.3%)	<0.001^a^

Abbreviations: AF, atrial fibrillation; CVA, cerebrovascular accident; MVR, mitral valve replacement; NYHA-FC, New York Heart Association functional class; re-PBMV, repeat percutaneous balloon mitral valvotomy; SR, sinus rhythm; Values were shown as mean±SD. ^a^*P* value is significant.

## Discussion

**Figure 1:**
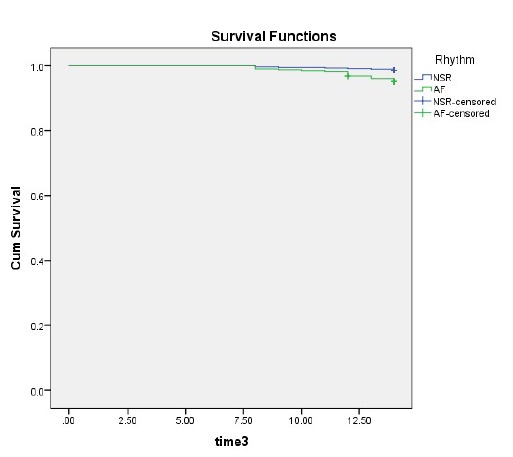
Kaplan-Meier survival curve shows survival time in patients with AF and SR.

The main findings of our study were that patients with AF who underwent PBMV had significantly lesser immediate success rates, higher mortality rate (Figure 1), higher mTMPG, lower MVA, higher short term and long term complication rates in comparison to patients with SR. Additionally, MS patients with AF before PBMV were significantly older, had higher mitral echocardiographic Wilkins scores, NYHA-FC, greater MR and mTMPGs compared to patients with SR.

These findings are in line with previous clinical, echocardiographic and hemodynamic findings of AF patients who were candidates for PBMV which showed a significant association between AF and older age, higher NYHA-FC, and higher mitral valve scores in MS patients.^10-12,14,15^ Similar to other studies, we also found that the results of PBMV in AF patients are poor compared to SR with increased short and long term adverse events.^12-15^ Leon et al showed that patients with AF had higher TMPG and smaller MVA before PBMV and higher mean LAP after PBMV.^12^ Fawzy et al found that patients with AF had smaller MVA and more re-stenosis after PBMV.^17^ Once more, Nair et al demonstrated that AF patients had a smaller MVA after PBMV compared to SR patients with higher rate of complications during follow-up.^18^ The association of AF with older age, higher NYHA-FC, higher mitral valve Wilkins score indicates that AF is a presentation of long term rheumatic MS with more severe morphological and structural changes which could estimate less desirable response to treatment and more incidence of adverse events.

The success rate of the PBMV procedure had different results in the previous studies. In the study by Maatouk et al, the success rate of the PBMV in AF vs. SR patients was statistically similar (89.7% vs. 92.3%, respectively; *P *= NS).^15^ On the other hand, Nair et al. had a similar results to our study, indicating a higher success rate of PBMV in SR patients (93.6% vs. 84.2%; *P *= 0.032).^18^ Different PBMV success rate in this study may related to the fact that our hospital is a tertiary referral center with the largest number of patients undergoing PBMV (N = 1800) that has ever been reported.

Our study had the greatest percentage of AF patients (41.5%) compared to previous studies (the next study being the one by Nair et al with 11.6% AF patients). The higher percentage of AF patients in our center does not necessarily represent a higher prevalence but more likely is caused by a referral bias since more complicated MS patients including patients with AF are referred to our tertiary centers.

We also found that some of the demographic and some baseline characteristics variables (high dyspnea NYHA-FC before PBMV in AF patients and lower MVA after PBMV in SR patients) can independently predict higher complication rates. Use of these variables in clinical decision making may reduce the rate of complications and improve the prognosis in these subsets of patients.

## Conclusion

Patients with MS who have AF are significantly older, have higher mitral valve scores, NYHA-FC, and greater MR and mTMPGs compared to patients with MS and SR. Additionally, after PBMV they have lesser immediate success rates, higher short term and long term complication rates, and higher mortality during follow up. It seems that earlier decision to PBMV could be justified to lesser AF and other complications related to duration of MS. Screening in endemic areas may have a role to earlier diagnosis and intervening to decrease AF and other adverse events which is related to chronicity of MS. Higher dyspnea NYHA-FC before PBMV in AF patients and lower MVA after PBMV in SR patients can independently predict higher adverse events.

Our findings support earlier intervention in patients with hemodynamically significant MS to decrease AF and AF related complications. PBMV is an acceptable low risk procedure in patients with AF despite of relatively lower success rate in comparison to patients with SR.

## Study limitations

About 800 patients who underwent PBMV at this center were excluded from the study due to lack of complete information requirements on their files for the study or follow-up information. Follow up duration and intervals were not identical in all patients. It is possible that patients with minimal symptom have not completed their follow up regularly. Also detailed information about the cause of death during follow up was not available.

## Ethical Approval

The study protocol was approved by the ethics committee of Tabriz University of Medical Sciences.

## Competing interests

Authors declare no conflict of interest in this study.

## Acknowledgments

The authors deeply appreciate all the staff of the Catheterization Laboratory of Shahid Madani heart center for their gracious assistance during this research.
